# Choice of bacterial DNA extraction method from fecal material influences community structure as evaluated by metagenomic analysis

**DOI:** 10.1186/2049-2618-2-19

**Published:** 2014-06-05

**Authors:** Agata Wesolowska-Andersen, Martin Iain Bahl, Vera Carvalho, Karsten Kristiansen, Thomas Sicheritz-Pontén, Ramneek Gupta, Tine Rask Licht

**Affiliations:** 1 Center for Biological Sequence analysis, Technical University of Denmark, Lyngby DK-2800, Denmark; 2 National Food Institute, Technical University of Denmark, Søborg DK-2860, Denmark; 3 Department of Biology, University of Copenhagen, Copenhagen N DK-2200, Denmark

## Abstract

**Background:**

In recent years, studies on the human intestinal microbiota have attracted tremendous attention. Application of next generation sequencing for mapping of bacterial phylogeny and function has opened new doors to this field of research. However, little attention has been given to the effects of choice of methodology on the output resulting from such studies.

**Results:**

In this study we conducted a systematic comparison of the DNA extraction methods used by the two major collaborative efforts: The European MetaHIT and the American Human Microbiome Project (HMP). Additionally, effects of homogenizing the samples before extraction were addressed. We observed significant differences in distribution of bacterial taxa depending on the method. While eukaryotic DNA was most efficiently extracted by the MetaHIT protocol, DNA from bacteria within the Bacteroidetes phylum was most efficiently extracted by the HMP protocol.

**Conclusions:**

Whereas it is comforting that the inter-individual variation clearly exceeded the variation resulting from choice of extraction method, our data highlight the challenge of comparing data across studies applying different methodologies.

## Background

In recent years, the community structure of human intestinal bacteria has received tremendous attention. The option of next generation sequencing for mapping of intestinal bacterial phylogeny and function has opened new doors to this field of research. However, little attention has been paid to the effects of sampling procedure and choice of methodology on the output resulting from such studies. Several practical challenges are associated with the collection of fecal samples in large human studies. Ideally, feces should be delivered anaerobically and processed directly after delivery. For obvious reasons, however, this normally cannot be achieved, and it is thus almost always necessary for microbiologists to base their studies on frozen samples that have been exposed to oxygen. Some reports indicate that freezing has a minor influence on the composition of extracted bacterial DNA from feces [1,2]. However, as long as we do not need to address the activities of live and oxygen-sensitive intestinal bacteria, but only to describe the composition of a given fecal bacterial community based on the bacterial DNA present in the sample, factors like oxygen exposure and freezing are not likely to have a large impact. It has thus previously been reported that, for example, the storage time of fecal samples before freezing does not have a major influence on the composition of fecal bacterial communities [3].

It is well documented that major differences exist between the mucosal and luminal bacterial populations of the human gut [4] and that the abundance and complexity of these populations vary between the different topographical sites of the bowel [5]. Keeping this in mind, it seems unlikely that the bacterial communities are completely evenly distributed within the volume of a fecal sample. Nevertheless, most recent studies of the human microbiota are based on DNA extraction from a very small subsample (typically 100 to 150 μl) of an un-homogenized sample. This is of little importance in cross-sectional studies where the inter-individual variations by far exceed variations attributed to subsample-site; however, it may be of major relevance in longitudinal studies comparing samples taken from the same individual over time. In the present study, we address the effect of homogenization versus subsampling from un-homogenized fecal material. To our knowledge, this has not previously been done.

The choice of DNA extraction method following sampling and storage probably also has an impact on the revealed community structure [6,7]. In particular, the first step of DNA extraction - disruption and/or lysis of the bacterial membranes - can be expected to be biased for specific bacterial taxa due to differences in cell wall structure and integrity. This step often involves bead-beating, a mechanical disruption of the bacteria, resulting in a higher yield of extracted DNA [6]. The most pronounced difference between bacterial envelopes is that between Gram-positive and Gram-negative cell walls. It has been shown that DNA from Gram-positive bacteria present in feces is more efficiently extracted if a sample has been frozen, probably because of the impact of freezing and thawing on the Gram-positive cell-wall, as bead-beating has a larger impact on the amount of Gram-positive DNA extracted from fresh samples compared to frozen samples [1]. Thus, most studies comparing methods of DNA extraction find that the major impact on the resulting measured community structure is caused by the use of bead-beating [1,6,8]. In the present study, comparable procedures for bead-beating are incorporated in both of the investigated methods, which are used by the two major research consortia, the American Human Microbiome Project (HMP) [9] and the European MetaHIT project [10]. These large collaborations have both resulted in many high-impact publications related to intestinal bacterial communities in humans [11-15]. Both take advantage of next generation sequencing, which is therefore also applied in the present study, in order to identify differences caused by sampling and DNA extraction.

## Methods

### Collection and preparation of fecal samples

**Figure 1:**
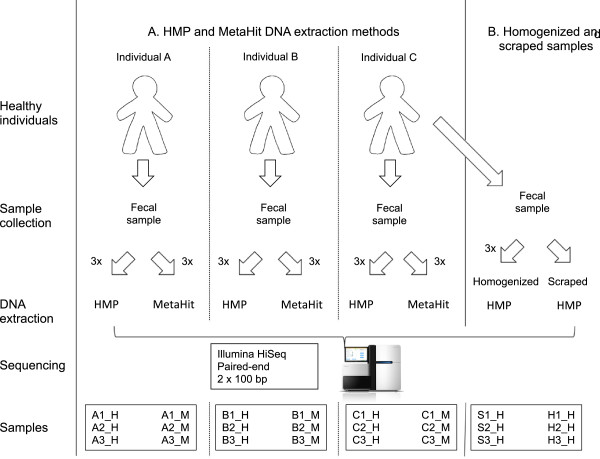
**Schematic representation of the study design. A**: Comparison of the HMP and MetaHIT DNA extraction methods, and **B**: comparison of homogenized and scraped samples.

For comparison of purification methods, approximately 50 g fecal samples were collected and processed from three healthy human volunteers within 4 hours. Samples not handled immediately were stored at 4°C. To each sample an equal volume of sterile milli-Q water was added and samples were homogenized using a Stomacher machine (2 times for 60 s at highest setting). Aliquots of 1 ml were then transferred to cryo-tubes and frozen at -80°C until DNA extraction by either the HMP or MetaHIT procedure as described below. For each procedure, three aliquots of each sample were purified, resulting in a total of 18 DNA extractions for next generation sequencing sequencing (Figure 1).To address the effect of homogenization, one fecal sample (approximately 15 g) was collected and processed immediately following two separate procedures representing small volume scrapings and homogenization. Initially, 200 mg scrapings were taken from the fecal sample at three different locations and transferred directly into the bead-beating solution of the Mobio PowerLyzer™ PowerSoil® DNA isolation Kit (MO BIO Laboratories, Carlsbad, CA, USA.). Then the remaining sample was homogenized in equal volume sterile milliQ water as described above and aliquots where transferred to 1 ml microcentifuge tubes. Three scrapings and three aliquots of the homogenized sample were purified by the HMP method as described below, resulting in a total of six DNA extractions for next generation sequencing (Figure 1).

### DNA purification by the MetaHIT method

Fecal slurries (1:1 feces/water) were thawed and centrifuged at 13,000 RPM for 10 minutes and the supernatant was removed. Approximately 200 mg (±10 mg) was transferred to a new 2 ml tube to which 250 μl guanidine thiocyanate and 40 μl N-lauryl sarcosine (10%) was added and allowed to stand for 10 minutes after which 500 μl N-lauryl sarcosine (5%) was added and the sample mixed by vortexing, centrifuged briefly and heat-treated at 70°C for 1 to 2 hours. To each tube 750 μl of zirconia/silica beads (0.1 mm) (BioSpec, number 11079101z, Bartlesville, OK, USA) were added and bead-beating was performed at 30 cycles/s for 5 minutes, followed by 10 minutes rest, and bead-beating again for 5 minutes (Retsch GmbH MM 300 mixer mill, Haan, Germany). The remaining extraction procedure followed the previously published procedure [16]. DNA concentrations were determined fluorometrically (Qubit® dsDNA BR assay, Life Technologies Europe, Naerum, Denmark) and purity was determined spectrophotometrically (NanoDrop 1000 Spectophotometer, Thermo Fisher Scientific, Waltham, MA, USA). Samples were stored at -20°C until sequencing.

### DNA purification by the HMP method

DNA was extracted from fecal samples using the Mobio PowerLyzer™ PowerSoil® DNA isolation Kit (MO BIO Laboratories) with slight modifications as follows. Fecal slurries (1:1 feces/water) were thawed and centrifuged at 13,000 RPM for 10 minutes and the supernatant was removed. Approximately 200 mg (±10 mg) was transferred to the bead-beating tube, bead solution added and then heat treated at 65°C for 10 minutes and then 95°C for 10 minutes. Additional heat treatment was also applied to samples undergoing the HMP procedure with the MoBIO DNA extraction kit. Bead-beating of the samples was performed at 30 cycles/s for 5 minutes, followed by 10 minutes rest, and bead-beating again for 5 minutes (Retsch MM 300 mixer mill); the beads in the kit were the same size as those used in for MetaHIT method. The remaining DNA extraction procedure followed the standard protocol supplied by the company and final elution of DNA was performed with 100 μl Tris (MoBIO buffer C6). DNA concentrations and purity were determined as stated above, and samples were stored at -20°C until sequencing.

### DNA library construction and sequencing

DNA libraries were pooled in groups of six samples per sequencing lane. Sequencing was performed with 100-nucleotide-long paired-end reads on the Illumina HiSeq 2000 (Illumina Inc., San Diego, CA, USA) sequencer with a total of four sequencing lanes containing the pooled libraries. Raw reads were submitted to the Short Read Archive (SRP040956).

### Read mapping

The total number of raw reads was downsampled to 29,012,054 reads per sample for samples used for comparison of extraction methods, and to 25,903,352 reads per sample for samples used to address the effect of homogenization. The final number of raw reads for each sample corresponded to the total number of raw reads obtained for the sample with the smallest number of reads within each group. The sequencing adaptors and any overrepresented sequences detected by FastQC [17] were removed and the high quality reads obtained from sequencing were trimmed with Trimmomatic [18]. The sequencing reads for each sample were then mapped to the reference human genome build 37 (GRCh37) using Burrow-Wheelers Aligner [19] to remove reads of human origin. In the mapping, the median insert size was estimated to be 164, and the average fragment length was 360 nucleotides. The reads that did not map to the human genome were mapped further to a set of reference sequences of known bacterial, fungal, plant and viral genomes retrieved from the NCBI Genome database (2 July 2012). The sequencing reads were also mapped to the assembled bacterial sequence catalogs generated by the HMP and the MetaHIT consortium, as well as to the gene catalog created for the purpose of this study, as described below.

Taxonomic abundance profiles were estimated for each sample with the MOCAT pipeline [20] incorporating bacterial references from the RefMG.v1 database [21], based on single copy marker genes from 1,753 bacterial reference genomes.

### Gene prediction

Gene catalogs for each sample were created using the MOCAT pipeline [20], starting with the downsized numbers of raw reads for each sample as described above. Briefly, the pipeline performs quality control of the raw reads, removes human contamination by mapping to the reference human genome, assembles the reads and predicts protein-coding genes on the assembled metagenomes. The redundancy within the resulting gene catalogs was further reduced with CD-HIT [22] using 90% sequence similarity and word size of five. Direct comparison of the individual gene catalogs was performed using CD-HIT at 90% sequence similarity. For the purpose of creation of the rarefaction curve of recovered genes as a function of raw sequencing reads, a complete gene catalog was created for all the samples. The raw reads were mapped against this complete gene catalog, and gene recovery for different numbers of reads was calculated from the resulting SAM file and plotted in the form of a rarefaction curve.

### Taxonomic and functional assignment

Taxonomic assignment for the method-specific genes was performed using BLAST + with the NCBI nucleotide database. Functional assignment for predicted genes was performed with BLAST + [23] against the eggNOG protein sequence database [24].

### Statistical analysis and cluster analysis

All statistical analyses were performed in R [25]. Statistical significance of the effect of the DNA extraction method on the observed abundances of bacterial genera were calculated with two-way ANOVA, and the differences between homogenized and scraped samples with Wilcoxon rank test. For multiple comparisons the *P*-values were corrected by Bonferroni correction and corrected *P*-values below 0.05 were considered statistically significant. Hierarchical clustering was performed with the heatmap2 package implemented in R. Plots were generated using ggplot2 and qplot R packages.

## Results

### Yield

Both the extraction methods resulted in sufficient yields and purity of DNA to perform Illumina HighSeq sequencing (Additional file 1). We observed significantly higher yields of DNA following extraction by the MetaHIT method compared with the HMP method (*P* < 0.0001, Mann-Whitney test), which may in part be caused by a limited binding capacity of DNA in columns used in the HMP method.

### Distribution of taxa resulting from the two methods

**Figure 2:**
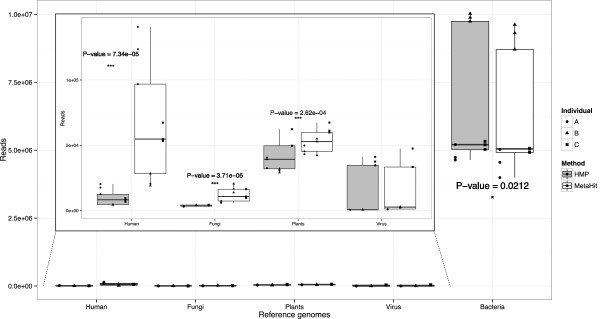
**Summary of read mapping to known reference genome sequences of different taxonomic groups.** As expected, the highest numbers of reads could be mapped to known bacterial genomes, with a slightly higher number of reads mapped with the HMP method (*P* = 0.0212). Despite overall low number of reads mapped to eukaryotic organism reference genomes, the differences in read counts attributed to the DNA extraction methods were highly significant, with the MetaHIT method resulting in a higher number of reads of eukaryotic origin in all cases. *P*-values were calculated with two-way ANOVA.

**Figure 3:**
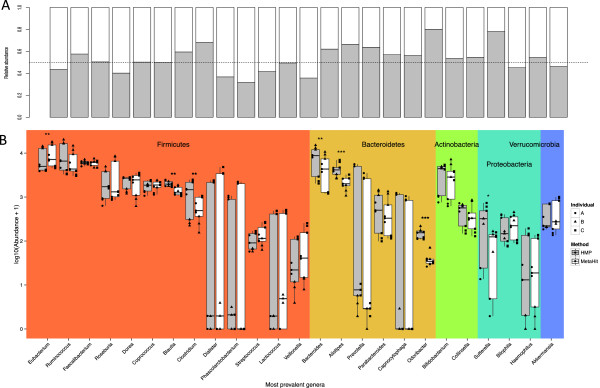
**Comparison of abundance estimations for bacterial genera with MetaHIT and HMP methods. (A)** Relative abundance of the most prevalent bacterial genera with MetaHIT and HMP DNA extraction methods. The horizontal line is plotted at a value of 0.5, corresponding to equal abundance of a given genus detected by both methods. **(B)** Estimated abundance of the 25 most abundant bacterial genera with established taxonomy mapping shows clear differences for several genera. The most significant differences are observed for Bacteroidetes; for three out of six cases the HMP method resulted in higher numbers of reads mapped to respective species. (*:*P* < 0.05; **:*P* < 0.01; ***:*P* < 0.001).

**Figure 4:**
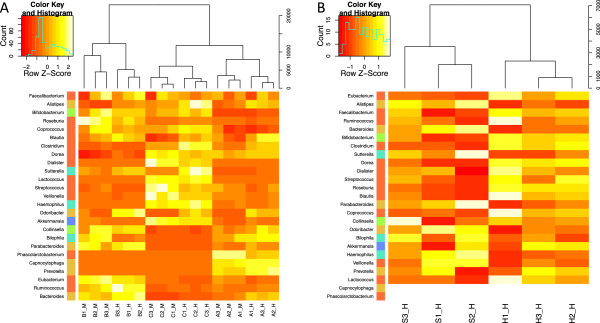
**Hierarchical clustering. (A)** Read counts mapped to the 25 most abundant bacterial genera for the three samples with three replicates for the two DNA extraction methods. Overall, the biggest observable difference results from between-sample variability, and regardless of the DNA extraction method used, the bacterial abundance profile can be assigned to the right individual. **(B)** Read counts mapped to the 25 most abundant bacterial genera for comparison of the effect of homogenizing and scraping of the samples. All the replicates were extracted from the same biological sample with the same DNA extraction method (HMP); therefore, the only source of variation comes from homogenizing or scraping of the samples before DNA extraction. As shown by the branch length in the sample clustering dendrogram, we observe higher between-replicate variability in the case of scraped samples.

Depending on the applied DNA extraction method, we observed significant differences in numbers of raw sequencing reads mapped to known reference genomes. Eukaryotic genomes of human, fungi and plants were significantly more present in the samples extracted with the MetaHIT method, while known bacterial genomes had significantly more reads mapped to them when extracted with the HMP method (Figure 2). Those differences were further examined for lower taxonomic ranks of bacteria, and we observed significant differences for several of the most abundant genera within the Bacteroidetes, Firmicutes and Proteobacteria phyla (Figure 3). The most prominent differences were observed among Bacteroidetes, where the HMP extraction method resulted in significantly higher estimated abundances for three out of the six most prevalent genera. This significantly (*P* = 0.00021) influenced estimation of the ratio between the two most common phyla, Firmicutes and Bacteroidetes (Figure S1 in Additional file 2).Hierarchical clustering of samples and bacterial genera based on their estimated abundance showed that the between-sample variation was higher than the between-method variation (Figure 4A). However, samples extracted with the same DNA extraction method clustered together, highlighting the impact of the method used on the detected composition of the sample.

### Effect of homogenization

Homogenization of samples before DNA extraction resulted in less within-sample variability, as evidenced by longer branch lengths for the non-homogenized samples in hierarchical clustering of the estimated abundance for the 25 most prevalent bacterial genera (Figure 4B). Additionally, the taxonomy abundance profiles cluster according to whether or not homogenization was performed. Due to the low numbers of samples compared in this case, any observed differences in taxonomy, function, number of mapped reads or number of predicted genes per replicate lack strict statistical significance, and are therefore not presented in detail in this work.

### Comparison of gene catalog composition

**Figure 5:**
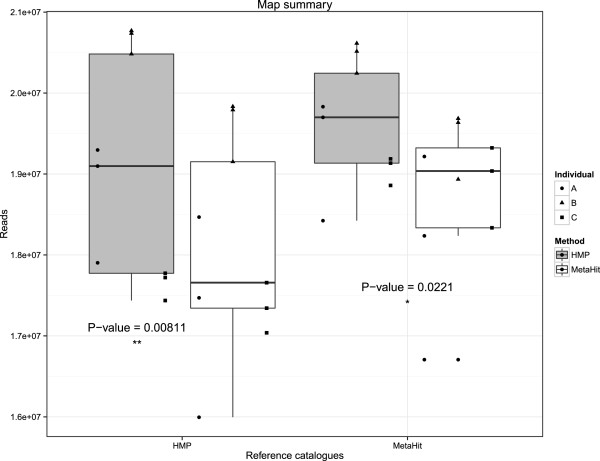
**Read mapping to the human gut microbiome reference sequence catalogues.** We observe significantly higher numbers of raw sequencing reads mapping to both reference catalogs for DNA extracted with the HMP method. For both extraction methods, more reads mapped to the MetaHIT catalog, suggesting that this catalog serves as a more complete representation of the gut microbiome. (*:P < 0.05; **:P < 0.01).

**Figure 6:**
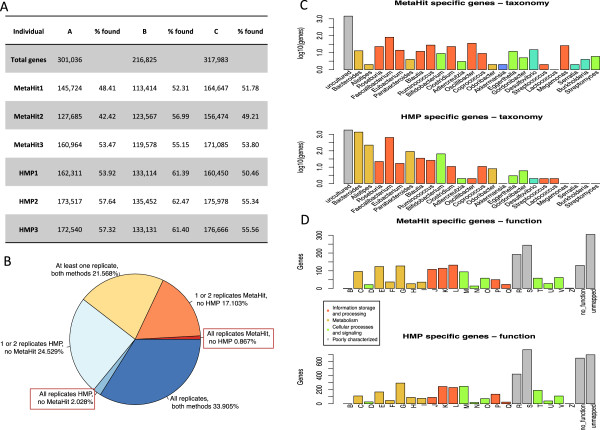
**Comparison of the gene catalog for different replicates of the two DNA extraction methods. (A)** For each individual a total gene catalog was created using all six replicates, resulting in total number of genes shown in the second row. The following rows show the numbers and percentage of the genes from the total gene catalog present in the gene catalogs for the individual replicates. **(B)** Schematic representation of the total gene catalog for one of the studied individuals, showing overlap of the genes discovered by all, some or none of the replicates with the HMP and MetaHIT methods. **(C)** Taxonomy annotation at the genus level for the genes specific to each method, that is, detected in all three replicates from one method, but none of the replicates from the other method. **(D)** Functional category annotation for the genes specific to each method.

**Figure 7:**
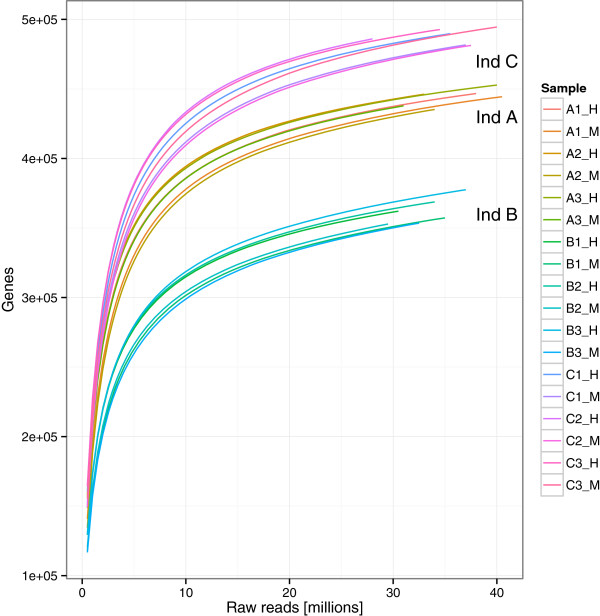
**Rarefaction curve.** Rarefaction curve created for the purpose of this study and showing the number of genes from the total gene catalog that could be discovered with increasing numbers of raw sequencing reads. The rarefaction curves for each individual group together in the plot, showing much more variation in gene content between individuals than variation attributed to the DNA extraction method.

The reference gene catalogs created by both the HMP and MetaHIT consortiums had significantly more reads mapped to them when extracted with the HMP method (Figure 5). Additionally, comparison of the number of predicted genes resulting from each DNA extraction showed that the HMP method yielded a significantly higher number of predicted genes (*P* = 0.0031) than the MetaHIT method (Figure 6A). A considerable amount of variability in the gene compositions was detected even between different replicates of the same extraction method, and only 33.9% of the total gene catalog for one sample was detected unanimously in all three replicates of both methods (Figure 6B).Exploration of both the taxonomy and functional category assignments of the genes detected in all three replicates of one method but in none of the replicates of the other method further highlights the differences in composition of the samples extracted with the different methods. More genes were detected in all replicates by the HMP method but in none by the MetaHIT method, than the reverse observation (2.0% versus 0.87%; Figure 6B). Differences were apparent for most genera (Figure 6C), and were also reflected in the functional categories of the mapped genes, with the most pronounced differences occurring in the functional categories B (chromatin structure and dynamics), J (translation, ribosomal structure and biogenesis) and O (post-translational modification, protein turnover, chaperone functions). The HMP extraction method also resulted in more genes with no function assigned (Figure 6D).Comparison of number of genes recovered from a total gene catalog created for the purpose of the present study (Figure 7) illustrated that, with the sequencing depths achieved, we captured a substantial proportion of the gene diversity in the studied samples, and that there were big differences in gene number between individuals. Regardless of the DNA extraction method used, however, the overall numbers of genes detected were roughly similar for each individual.

## Discussion

We have applied next generation sequencing of the fecal metagenome to address differences between the two procedures chosen for DNA purification by the two major research consortia, MetaHIT and HMP. The first observation was that the MetaHIT protocol, which is based on laboratory-made buffers and solutions, resulted in a significantly higher yield than the kit-based HMP protocol (Additional file 1); however, yield and purity of DNA extracted with both protocols were sufficient for Illumina-based deep metagenome sequencing. These factors are crucial to consider before choosing a method, as both yield and purity will affect the applicability of the DNA for next generation sequencing. However, if many samples are to be analyzed, the extra amount of labor affiliated with the MetaHIT protocol may also be a relevant factor. We anticipated that the approach of the MetaHIT consortium might also lead to less reproducibility (larger technical variation) in the data than the standardized, kit-based approach used by HMP, but this was not indicated by the data. Still, as all our extractions were performed in the same lab and by the same person, it cannot be excluded that a larger amount of between-lab and between-person variation may result from applying a non-kit-based protocol.

In spite of comparable bead-beating steps applied in the two methods, we found significant differences between the measured community structures resulting from DNA extracted with each of the procedures. Most striking was the observation that a significantly higher amount of eukaryotic DNA (humans, fungi, plants) was extracted using the MetaHIT protocol compared with the HMP protocol (Figure 2). Conversely, we observed significantly higher numbers of reads mapping to bacterial gene catalogs from HMP as well as MetaHIT consortia for DNA extracted using the HMP protocol (Figure 5). We speculate that this might be because the lysis procedure of the MoBio® kit used by the HMP consortium may be optimized to lyse bacterial and not eukaryotic envelopes. When focusing on the 25 most abundant genera, we observed that, with only a few exceptions, the MetaHIT method estimated a lower abundance of the genera within the Bacteroidetes than the HMP method (Figure 3A). For three out of six Bacteriodetes, the estimated abundances were significantly lower when applying the MetaHIT protocol (Figure 3B). This was also reflected in a significant difference between the Firmicutes/Bacteroidetes ratio obtained with the two methods (Figure S1 in Additional file 2). This ratio is important for the interpretation of the functional capacity of the intestinal metagenome, and has been proposed to be of importance for risk of obesity [26,27]. None of the most abundant species within the phyla Actinobacteria and Verrucomicrobia were differently affected by the two methods, indicating that the impact of the extraction methods on Firmicutes and Bacteriodetes are not solely due to the differences between Gram-positive and Gram-negative bacterial membranes. In general, however, the Gram-negative genera were most sensitive to choice of method, as one of the proteobacterial genera was also differently affected, with the highest abundance of *Sutterella* obtained after HMP protocol extraction. Taken together, only 3 out of 16 Gram-positive genera were differently extracted, while this was the case for 4 out of 9 Gram-negative genera, which were all most efficiently extracted by the HMP protocol. Although speculative, it is likely that for the Gram-negative cell envelopes, which are generally easier to disrupt than Gram-positive cell walls, differences between membrane structure of the individual species play a more pronounced role in their susceptibility to the lysis approaches applied. We find particularly that the systematic differences in extraction of DNA from Bacteroidetes species are important to consider when comparing data across studies where different protocols have been applied. In studies where it is important to detect low-abundant species within this phylum, it may be considered to apply the HMP protocol, which seemed to extract DNA from this particular phylum more efficiently.

Although the listed differences between protocols are important to consider, we found it reassuring that the variation attributed to the choice of method was still less than the variation attributed to differences between individual samples (Figure 4A; Figure S2 in Additional file 2). We also observed that the overall correlation of the two methods in their capacity to detect even low abundant bacterial species was very high (rho = 0.97), despite a skew towards more Bacteroidetes species detected with the HMP method (Figure S3 in Additional file 2). However, a similar skew was not seen for gene abundance (Figure S4 in Additional file 2). Comparison of gene catalogs for each individual replicate showed large variation in the gene content detected with each individual DNA extraction, and only approximately 34% of the total gene catalog was detected within all three replicates of both methods (Figure 6B). We observed a number of 'method-specific' genera and genes (Figure 6C,D), and a more careful examination of these revealed that the HMP protocol clearly enriched for genera within the Bacteroidetes.To our knowledge, this is the first study addressing the effect of homogenization of fecal samples on the variability of metagenomic data. Not surprisingly, we found that homogenization of samples before DNA extraction resulted in less within-sample variability (Figure 4B). Although this is probably of little relevance in cross-sectional studies based on a ‘snapshot’ of human populations to be compared, it is worth considering in longitudinal studies, where samples taken from the same individual are to be compared with each other. However, in studies addressing the abundance of, for example, bacterial mRNA, the homogenization procedure must be expected to affect the outcome, and is not recommended.

## Conclusion

We found a skew in both the taxonomic and functional distribution of genes specific to the DNA extraction method used, and those differences might have an influence on the functional interpretation of results, even though they overall affect a small percentage of the total estimated microbial communities. In this context, it should be noted that the sequencing approach [28], the sequencing technology [29,30] and the choice of bioinformatics tools [31] also affect the outcome of metagenomic studies, although these issues were beyond the scope of the present study. Furthermore, current interpretation of metagenomic results is limited and defined by previously characterized and cultured bacterial species. While the vast majority of the bacterial genes present in the human gut remain unclassified in terms of taxonomy and function, defining the impact of our ‘other genome’ on human health and disease is thus still a challenging task.

### Consent

Written informed consent was obtained from the three healthy volunteers for the publication of this report.

## Abbreviations

HMP: Human Microbiome Project; MetaHIT: Metagenomics of the Human Intestinal Tract.

## Competing interests

The authors declare that they have no competing interests.

## Authors’ contributions

AWA: data collection and analysis, manuscript writing and final approval of the manuscript. MIB: conception and design, data collection and analysis, manuscript writing and final approval of the manuscript. VC: help with data analysis, critical revision and final approval of the manuscript. KK: conception, critical revision and final approval of the manuscript. TSP: conception and design, critical revision and final approval of the manuscript. RG: conception and design, critical revision and final approval of the manuscript. TRL: conception and design, manuscript writing and final approval of the manuscript. All authors read and approved the final manuscript.

## Authors’ information

KK, TSP and RG are part of the MetHIT consortium.

## Supplementary Material

Additional file 1: Table S1 DNA yield and purity obtained with the two methods. Click here for file

Additional file 2: Figure S1 Firmicutes/Bacteriodetes ratio obtained with the two methods. **Figure S2.** Heatmap (as Figure 4A) based on all taxonomic groups. **Figure S3.** Correlation of bacterial abundances obtained by the two methods. **Figure S4.** Correlation of gene abundances obtained by the two methods. Click here for file
