# Supplementary material for: Choice of bacterial DNA extraction method from fecal material influences community structure as evaluated by metagenomic analysis
Source: Microbiome. 2014 Jun 5;2:19. doi: 10.1186/2049-2618-2-19 (PMC4063427; doi:10.1186/2049-2618-2-19)
Supplement: Additional file 1: Table S1 — DNA yield and purity obtained with the two methods. [file 2049-2618-2-19-S1.pdf]

Additional Table 1

Sample name	Concentration (ng/μl) (mean ± SD)	OD_{260}/OD_{280} (mean ± SD)	OD_{260}/OD_{230} (mean ± SD)
A_H	46 ± 12	1.95 ± 0.03	1.41 ± 0.06
B_H	21 ± 5	1.96 ± 0.12	0.93 ± 0.22
C_H	30 ± 5	1.89 ± 0.06	1.06 ± 0.20
A_M	91 ± 8	1.08 ± 0.03	1.10 ± 0.14
B_M	105 ± 4	1.01 ± 0.05	1.16± 0.09
C_M	234 ± 38	1.38 ± 0.05	1.74 ± 0.17
S_H	23 ± 8	2.09 ± 0.36	0.80 ± 0.05
H_H	48 ± 4	1.85 ± 0.02	1.30 ± 0.31

In sample names, the first letter (A,B or C) refers to a given individual, while the second letter refers to the DNA extraction method applied by HMP (H) or MetaHit (M). First letters S and H refer to scraped or homogenized samples from one individual. In all cases DNA was extracted from fecal samples in triplicate. Concentrations were determined by Qubit fluorescence assay and OD_{260}/OD_{280} and OD_{260}/OD_{230} ratios were determined by Nanodrop analysis.
