# Supplementary figures and images for: Choice of bacterial DNA extraction method from fecal material influences community structure as evaluated by metagenomic analysis
Source: Microbiome. 2014 Jun 5;2:19. doi: 10.1186/2049-2618-2-19 (PMC4063427; doi:10.1186/2049-2618-2-19)

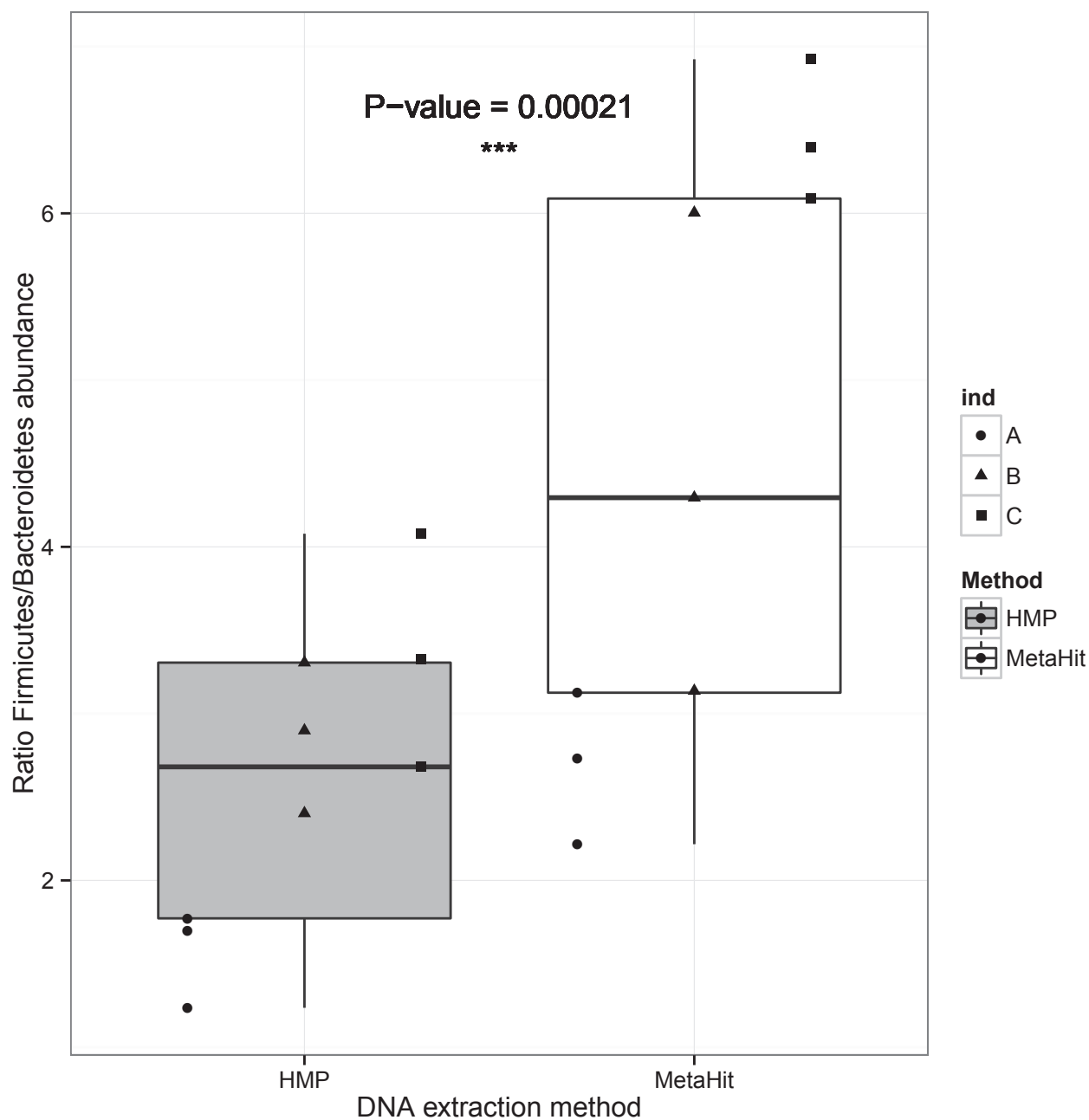

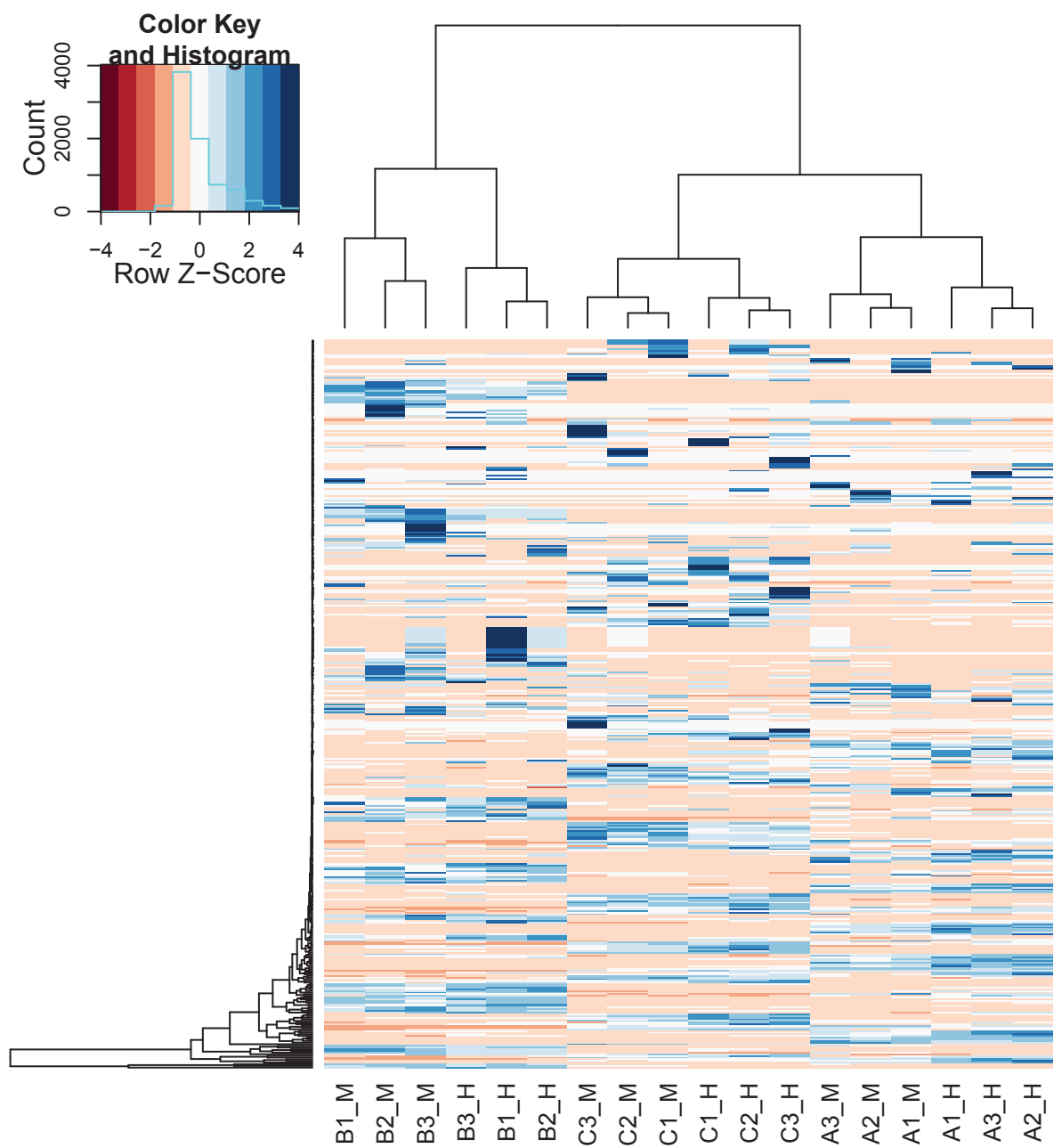

Correlation of bacteria abundance  
 $\rho = 0.97$

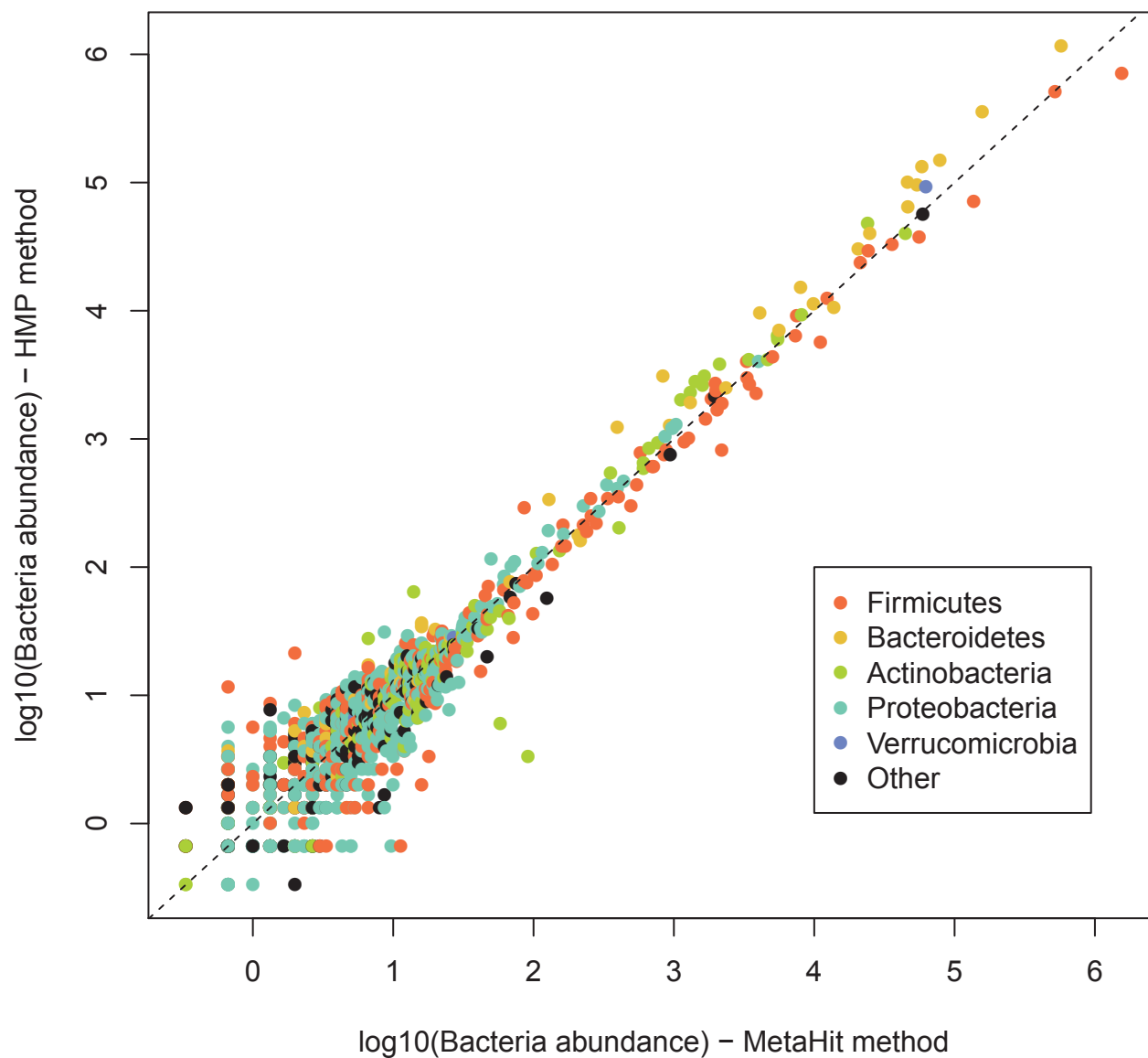

Correlation of gene abundance – Individual A  
 $\rho = 0.94$

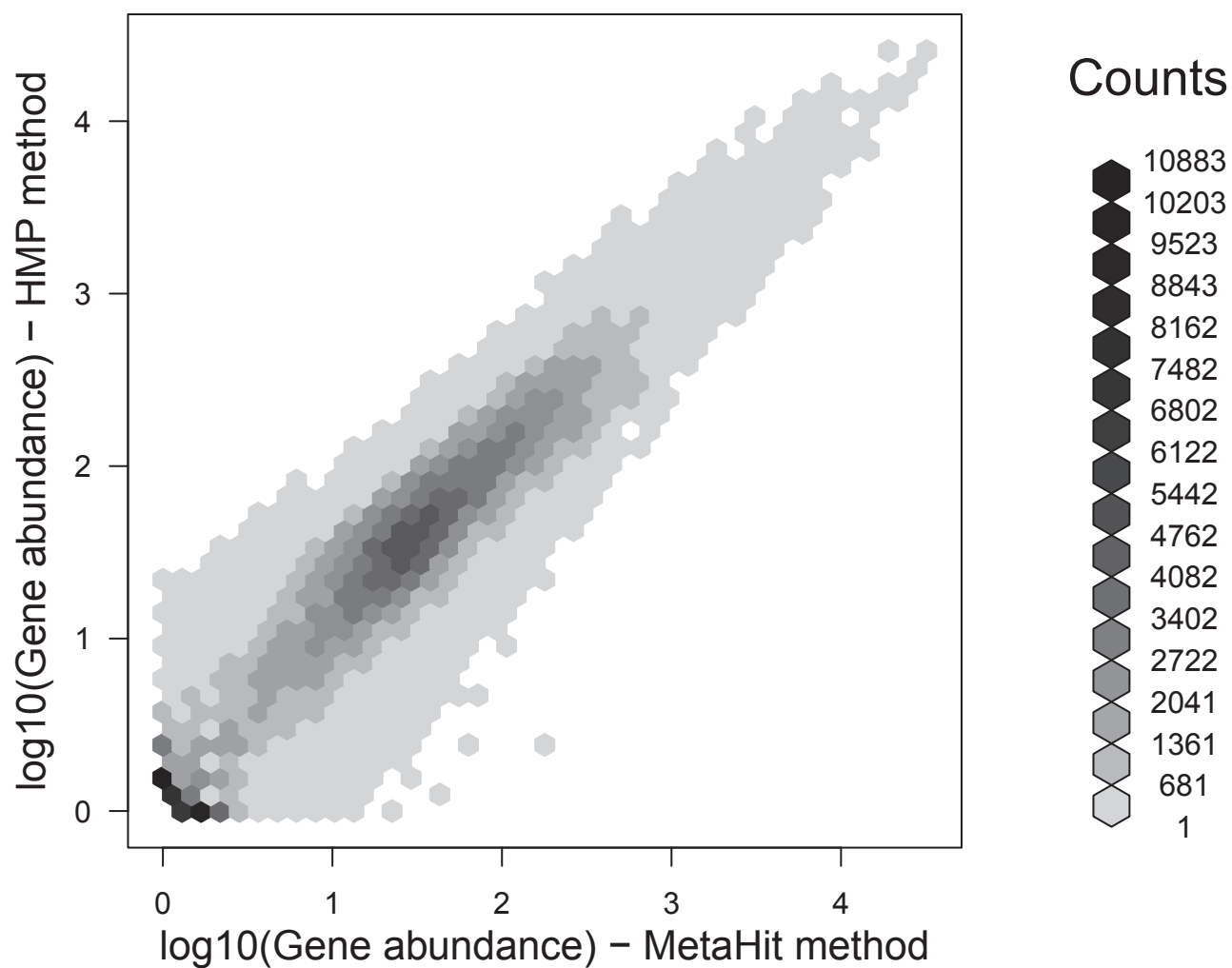

Supplement: Additional file 2: Figure S1 — Firmicutes/Bacteriodetes ratio obtained with the two methods. Figure S2. Heatmap (as Figure 4A) based on all taxonomic groups. Figure S3. Correlation of bacterial abundances obtained by the two methods. Figure S4. Correlation of gene abundances obtained by the two methods. [file 2049-2618-2-19-S2.pdf]
